# p38 Signalling Pathway

**DOI:** 10.3390/ijms22031003

**Published:** 2021-01-20

**Authors:** Juan José Sanz-Ezquerro, Ana Cuenda

**Affiliations:** 1 Department of Molecular and Cellular Biology, Centro Nacional de Biotecnología/CSIC (CNB-CSIC), Campus-UAM, 28049 Madrid, Spain; 2 Department of Immunology and Oncology, Centro Nacional de Biotecnología/CSIC (CNB-CSIC), Campus-UAM, 28049 Madrid, Spain

## Editorial on the Special Issue: p38 Signalling Pathway

p38 Mitogen activated protein kinases (p38MAPK) are a highly evolutionary conserved group of protein kinases, which are central for cell adaptation to environmental changes as well as for immune response, inflammation, tissue regeneration, and tumour formation. p38MAPKs are Ser/Thr kinases that catalyse the reversible phosphorylation of proteins in response to different stimuli such as cellular stress, infections or cytokines. This kinase family is composed by four members encoded by different genes: p38α (*MAPK14*), p38β (*MAPK11*), p38γ (*MAPK12*), and p38δ (*MAPK13*). p38α is the best characterized, whereas the functions of p38β, p38γ and p38δ have been less studied. p38α was the first p38MAPK identified by four different laboratories as a 38 kDa polypeptide that underwent tyrosine phosphorylation in response to endotoxin treatment, cytokines or cellular stress [1]. Some years later, other three p38MAPK isoforms were identified, p38β, p38γ (also known as ERK6 or SAPK3) and p38δ (initially called SAPK4). The four p38MAPKs are ubiquitously expressed, although the levels of expression of each isoform varies in specific tissues; for example, p38β is abundant in brain, whereas p38γ is highly expressed in skeletal muscle, and p38δ in endocrine glands, testis, pancreas, kidney and small intestine [1,2].

The activation of p38MAPK is mediated by a cascade of kinases, which become sequentially activated in response to a wide range of stimuli. This cascade is typically organized in three tiers. p38MAPKs are activated by the MAPK kinases (MAP2K or MKK) MKK3 and MKK6 and, in the case of p38α, also by MKK4, by dual phosphorylation of the Thr-Gly-Tyr motif in the activation loop. The MAP2Ks are in turn activated by phosphorylation by various MAP2K kinases (MAP3Ks) depending on the stimulus and cell context [1,2]. It has been shown that the activation of p38α can also be regulated by MAP2K-independent mechanisms such as for example, autophosphorylation mediated by interaction with TAB1 (transforming growth factor β-activated protein kinase 1 (TAK1) binding protein 1) in cardiomyocytes, or by ZAP70-mediated phosphorylation at Tyr323 in T cells [1]. Based on previous published results showing that protein arginine methyltransferase 1 (PRMT1) promotes p38α activation in the differentiation of erythrocytes, in this special issue, Liu et al. provide new insight into the complexity of p38MAPK regulation by identifying a new molecular mechanism of PRTMT1-modulation of p38α activation [3]. They show that methylation of p38α at Arg49 and Arg149 by PRMT1, increased its interaction with the upstream activator MKK3 (but not MKK6) and with the downstream substrate MAPK activated protein kinase 2 (MAPKAPK2 or MK2). Liu et al. also show that p38α methylation positively regulated AraC-mediated erythroid differentiation and suggested that these findings extend the possible ways of p38MAPK pathway intervention, other than kinase activity inhibition [3].

p38MAPKs control multiple biological functions in the cell by phosphorylating numerous protein substrates, both in the cytoplasm and in the nucleus. p38MAPKs can translocate to the nucleus, either in resting cells or after stimulation. Maik-Rachline et al. describe some of the mechanisms involved in the nucleo-cytoplasmic shuttling of p38α and p38β (p38α/p38β), which involve the binding to several importins (Imp7, 9 and 3), and to Nuclear Export Signal (NES)-containing substrates (such as MK2) for their export back to the cytoplasm [4]. In their article, the authors also discuss how prevention of p38α/p38β nuclear translocation could be a good tool to prevent some inflammatory diseases, or even cancer like colorectal cancer [4].

The interest in the group of p38MAPKs has grown continually since their discovery. Studies using genetic and pharmacological tools have provided information on the function of these kinases and show that they have a big impact not only on the coordination of the cellular responses to nearly all stressful conditions, but also on the development of prevalent pathologies related to inflammation, autoimmune diseases, neurodegeneration or cancer, among others. Since p38α is the most studied among the p38MAPKs, over the past two decades this kinase has been suggested as a potential therapeutic target for the treatment of different pathologies, particularly inflammatory diseases and cancer [5]. It has been shown that p38α plays opposing effects, either as tumour suppressor or promoter, depending on different types of cancer or on phases of cancer development [6]. Recently, the implication of other p38MAPK (p38γ and p38δ) in tumour development has been studied; however, the role of p38β is still unclear. Roche et al., compile the current knowledge about the involvement of p38β in regulating oncoproteins that contribute to tumour initiation, progression, angiogenesis and metastases; or even in other aspects related to cancer disease such as cachexia or pain [7].

Idiopathic pulmonary fibrosis (IPF) is a lung pathology in which inflammation and formation of fibrotic foci are determinant for disease progression and patient mortality. Matsuda et al., examine the implication of p38MAPK activity in bleomycin-induced IPF by performing a comparative transcriptome analysis in alveolar epithelial type II cells from wild type mice and mice expressing either constitutive active MKK6 as a gain-of-function model or dominant negative p38α as a loss-of-function model [8]. These authors found that enhanced p38 signalling in the lungs was associated with increased transcription of genes driving p38MAPK pathway and also correlated with increased bleomycin-induced fibrosis severity, indicating a role of this pathway in the progression of pulmonary fibrosis [8].

p38MAPKs play a pivotal role in muscle physio-pathology; thus, there are numerous evidence implicating them in skeletal muscle and cardiovascular system differentiation/development, in muscle metabolism, as well as in cardiovascular mortality [1,2]. Romero-Becerra et al., revise the role of all p38MAPK isoforms in cardiomyocyte differentiation and growth, and discuss how they are involved in pathological conditions related to ischemia-reperfusion injury, heart failure or cardiac arrhythmia [9]. Bengal et al., focus on p38MAPK implication in glucose metabolic adaptation of skeletal muscle to exercise and obesity; they also discuss p38MAPK role in pathological conditions leading to type II diabetes and the possibility of targeting these kinases in therapies for diabetes treatment [10].

Other processes in which p38MAPK are central elements are those leading to different neuropathologies; this is studied and discussed in several articles of this special issue. Hu et al., report a novel mechanism of kainic acid (KA)-induced seizure that involves p38-mediated phosphorylation at Thr607 of the ion channel Kv4.2, which modulates hippocampal neuronal excitability and seizure strength. Importantly, the authors also show that pharmacological inhibition of p38α reduced neuronal excitability and diminished seizure intensity, indicating the importance of targeting this kinase to mitigate seizure severity in neurological disorders like epilepsy that affect a vast amount of the world population [11]. During the last decade different members of the p38MAPK pathways have joined the group of signalling pathways involved in the development of neurodegenerative diseases, such as Alzheimer’s disease. In this context, Germann & Alam review the connection between the Ras-related protein Rab5 (an endosome-associated protein implicated in neurodegenerative disease development) and p38α [12]. Since p38α regulates RAB5 activity, the authors also discuss how brain-penetrant selective p38α inhibitors will provide significant therapeutic advances in neurogenerative disease through normalizing dysregulated Rab5 activity [12]. Falcicchia et al., summarize the role of p38MAPKs, in particular of p38α, in the regulation of synaptic plasticity and its implication in an animal model of neurodegeneration; and extensively describe the use of specific inhibitors for improving synaptic and memory deficit in Alzheimer’s disease mouse models [13]. Finally, to shape the final outcome of this Special issue, El Rawas et al. nicely review and discuss the function of p38α in stress, anxiety, depression, and in the rewarding effect of drugs of abuse, particularly cocaine [14].

In this Special Issue, we wished to offer a platform for high-quality publications on the latest advances on p38MAPK pathway substrates, functions and regulation; the mechanisms underlying the role of p38MAPK in different pathologies; and therapeutic opportunities associated with modulation of p38MAPK activity. We expect that the information collected in this issue will be of interest to researchers working in signalling, and stimulate them to continue in their efforts to increase our knowledge on this exciting p38MAPK field. Finally, we would like to thank all researchers who dedicated their effort and time to write their articles for this Special Issue; and specially to the reviewers that generously gave their time and expertise in the field to improve the quality of the published articles.
